# Author Correction: Time reversed optical waves by arbitrary vector spatiotemporal field generation

**DOI:** 10.1038/s41467-021-20944-8

**Published:** 2021-01-18

**Authors:** Mickael Mounaix, Nicolas K. Fontaine, David T. Neilson, Roland Ryf, Haoshuo Chen, Juan Carlos Alvarado-Zacarias, Joel Carpenter

**Affiliations:** 1 School of Information Technology and Electrical Engineering, The University of Queensland, Brisbane, QLD 4072 Australia; 2 Nokia Bell Labs, 791 Holmdel Road, Holmdel, NJ 07722 USA

**Keywords:** Ultrafast photonics, Optical physics

Correction to: *Nature Communications* 10.1038/s41467-020-19601-3, published online 16 November 2020.

The original version of this Article contained an error in the caption of Figure 3, which incorrectly read ‘**a** Spatiotemporal demonstration.’ The correct version states ‘Spatiospectral’ in place of ‘Spatiotemporal’. This has been corrected in both the PDF and HTML versions of the Article.

